# StatXFinder: a web-based self-directed tool that provides appropriate statistical test selection for biomedical researchers in their scientific studies

**DOI:** 10.1186/s40064-015-1421-9

**Published:** 2015-10-22

**Authors:** Aslı Suner, Gökhan Karakülah, Özgün Koşaner, Oğuz Dicle

**Affiliations:** Department of Biostatistics and Medical Informatics, School of Medicine, Ege University, 35100 Bornova, Izmir Turkey; Department of Medical Informatics, Health Sciences Institute, Dokuz Eylül University, 35340 Inciraltı, Izmir Turkey; Neurobiology-Neurodegeneration and Repair Laboratory, National Eye Institute, National Institutes of Health, Bethesda, MD 20892 USA; Department of Linguistics, Faculty of Letters, Dokuz Eylül University, 35160 Buca, Izmir Turkey; Department of Radiology, School of Medicine, Dokuz Eylül University, 35340 Inciraltı, Izmir Turkey

**Keywords:** Statistical test selection, Decision support, Biomedical research, Parametric statistical tests, Non-parametric statistical tests

## Abstract

The improper use of statistical methods is common in analyzing and interpreting research data in biological and medical sciences. The objective of this study was to develop a decision support tool encompassing the commonly used statistical tests in biomedical research by combining and updating the present decision trees for appropriate statistical test selection. First, the decision trees in textbooks, published articles, and online resources were scrutinized, and a more comprehensive unified one was devised via the integration of 10 distinct decision trees. The questions also in the decision steps were revised by simplifying and enriching of the questions with examples. Then, our decision tree was implemented into the web environment and the tool titled StatXFinder was developed. Finally, usability and satisfaction questionnaires were applied to the users of the tool, and StatXFinder was reorganized in line with the feedback obtained from these questionnaires. StatXFinder provides users with decision support in the selection of 85 distinct parametric and non-parametric statistical tests by directing 44 different yes–no questions. The accuracy rate of the statistical test recommendations obtained by 36 participants, with the cases applied, were 83.3 % for “difficult” tests, and 88.9 % for “easy” tests. The mean system usability score of the tool was found 87.43 ± 10.01 (minimum: 70—maximum: 100). A statistically significant difference could not be seen between total system usability score and participants’ attributes (p value >0.05). The User Satisfaction Questionnaire showed that 97.2 % of the participants appreciated the tool, and almost all of the participants (35 of 36) thought of recommending the tool to the others. In conclusion, StatXFinder, can be utilized as an instructional and guiding tool for biomedical researchers with limited statistics knowledge. StatXFinder is freely available at http://webb.deu.edu.tr/tb/statxfinder.

**Electronic supplementary material:**

The online version of this article (doi:10.1186/s40064-015-1421-9) contains supplementary material, which is available to authorized users.

## Background

Statistics, as a field of science, helps to obtain more valid and reliable interpretation of the results by the data analysis using latest tools of the field. Statistical methods are one of the most prominent tools used to facilitate the transformation of the data obtained in scientific research into knowledge. However, due to technical approaches, language and methodology options, difficulties are often experienced in the selection of the correct statistical tool and depending on these issues, incorrect applications of statistical methods are frequently seen in the data analysis phase of biomedical research (Twycross and Shields 2004; McCrum-Gardner 2008; Nyirongo et al. 2008; Okeh 2009; Jaykaran 2010; Nayak and Hazra 2011; Gunawardena 2011). These errors, generally, center on decision making in statistical test selection, sample size decision, clinical trial planning, reporting and interpreting statistical results, selection of control group, use of figures and tables, and obtaining false positive results following multiple testing (Lang 2004; Strasak et al. 2007; McCrum-Gardner 2008; Nyirongo et al. 2008; Harris et al. 2009; Charan and Saxena 2012). In addition, one of the frequent errors is the selection of an inappropriate test for the data type and data analysis that could lead to the incorrect interpretation of research findings and false conclusions resulting in expenditure of in vain researchers’ precious time and resources (Nyirongo et al. 2008; Harris et al. 2009).

Since the use of appropriate statistical test requires expertise thus statisticians are an integral part of research teams. However, the researcher may experience difficulties in accessing a statistician to analyze the data appropriately. In such a case, applying the decision trees for statistical test selection in the analysis of the research data can reduce statistical errors. Therefore, currently, there are various decision trees developed for the selection of appropriate statistical test (Gaddis and Gaddis 1990; Rosner 2000; Mertler and Vannatta 2002; Twycross and Shields 2004; Leeper 2006; McCrum-Gardner 2008; Okeh 2009; Jaykaran 2010; Marusteri and Bacarea 2010; Normando et al. 2010; du Prel et al. 2010; Nayak and Hazra 2011; Gunawardena 2011; Johnson and Karunakaran 2014; Bettany-Saltikov and Whittaker 2014). In these decision trees, the users are presented a series of questions and they are expected to answer these questions. The questions are related to the user’s data such as purpose of use, sample size, sample independency and variable type(s). An analysis method appropriate for the purpose of use and the data set is recommended in these decision trees according to answers given in the decision steps.

Since the decision trees are limited in number and provide decision support for only certain statistical tests, the researcher is required to determine the appropriate decision tree in addition to the appropriate test. For instance, while the tree structure prepared by Nayak and Hazra provides decision support for parametric and nonparametric methods, the decision tree of Mertler and Vannatta is appropriate for multivariate methods (Mertler and Vannatta 2002; Nayak and Hazra 2011). On the other hand, the tree developed by Gaddis and Gaddis renders it possible to select only nonparametric tests (Gaddis and Gaddis 1990). However, a unified and extensive tree structure for the selection of statistical tests that are commonly used and provided with decision support in the aforementioned decision trees in biomedical domain is not present yet. Moreover, the questions for the users in the present decision trees may be difficult to understand and too confusing for non-statistician users. In this respect, it is of great importance that an extensive decision tree, independent of the user’s background, with easily answered questions in the decision steps should be developed.

In recent years, the use of computer based tools for statistical test selection has become more frequent. Among these tools, Statistics Open For All (SOFA), a free and open-source standalone software, provides the researchers of different backgrounds with decision support for statistical data analysis (Johnson and Karunakaran 2014). SOFA asks users three questions in analyzing differences between groups: (1) the number of groups, (2) normality and (3) independence. It also asks two questions concerning the analysis of the relations between variables: (4) data type and (5) normality. It then recommends a limited number of parametric and non-parametric tests based on the answers to these five questions. The preferred method for statistical test selection in the dental field is a PowerPoint-based tool by Normando et al. This tool enables the selection of simple statistical tests by students and inexperienced researchers who would not be comfortable with more involved statistical analysis (Normando et al. 2010). A web-based decision tree devised by the UCLA Statistical Consulting Group includes questions about the number and the type of variables in the user data and recommends the test options that could be used in testing researchers’ hypothesis (Leeper 2006). In addition, UCLA’s tool provides detailed information on how the recommended statistical method(s) could be implemented in different statistical packages such as SPSS and R (Team RDC 2008; IBM SPSS 2012). In a similar fashion, there is a paid smartphone application developed for the selection of the appropriate statistical test by researchers in the field of life sciences and for use in the peer review process by the journal reviewers and editors (Wiles and Bishop 2013). Integration of computer-based decision making tools facilitates the otherwise complex process of statistical test selection.

In this study, presented initially at the Medical Informatics Europe 2014 Conference, we aimed at examining the currently available decision trees for statistical test selection in biomedical domain and forming a more extensive tree structure by overcoming the current deficiencies in these trees (Suner et al. 2014). In addition, we created a web-based decision support tool named “StatXFinder” to facilitate the selection of the appropriate test method for researchers while testing their hypotheses. Moreover, a questionnaire study was conducted for determining the usability and a user satisfaction of the tool. Some question items in the decision tree and statistical test recommendations were updated with regard to the user feedback, and the tool was then launched for public use on the Internet.

StatXFinder is available for access as a reasoning and decision support tool which aims to recommend the most appropriate statistical method with the least number of questions. In addition, this tool is designed to be instructional and to include extensive information about the basic statistical terms, explanations of methods, and selection justifications.

## Methods

### Integration of distinct decision trees for statistical test selection

An online manual search was conducted to determine the currently available decision trees regarding appropriate statistical test selection. As a result of the online search, the decision trees presented in textbooks, published articles, and online resources were examined in terms of their corresponding statistical tests. The statistical tests provided with decision support by all of the tree structures were included in the study, and the questions for users at each decision step of each decision tree were determined. Missing aspects of the tree structures were identified when no test could be provided for a set of user specifications.

After scrutinizing each decision tree, the most comprehensive one was selected and acknowledged as the base decision tree. Later, the tests not included in the base tree structure, but found in others were determined and were integrated to the base decision tree. As a result, the base decision tree was updated and modified to a more comprehensive one. In addition, the form of question items was reorganized in the modified and refined base decision tree and all questions were organized as yes–no questions.

After the structural changes in the base decision tree, most questions were rewritten to enhance the understandability of the decision tree steps for the users at the decision steps of the decision tree. At this stage, the questions were assessed separately, in terms of their understandability, in a panel comprising of four researchers from divergent research areas, including: bioengineering, biostatistics, linguistics, and radiology. If appropriate, some of the questions were reorganized and a brief explanation was written for each. In addition, basic statistical terms such as “variable”, “sample”, or “continuous” mentioned in some of the questions, were determined and the explanations of these terms were written using three different statistical glossaries (Sahai and Khurshid 2002; Cramer and Howitt 2004; Everitt and Skrondal 2010).

Five expert statisticians with experience in biostatistics from Dokuz Eylül University, Department of Statistics, and Ege University, Department of Biostatistics and Medical Informatics, gave their opinions during the development of the decision tree devised here. The unified decision tree structure, the questions and order of the questions in the decision tree were evaluated with these five experts. The experts in the panel were requested to examine each branch in the decision tree and to assess whether the tests recommended by the decision tree were compatible with the answers given.

### Development of a web-based decision support tool

In order to facilitate the access of potential end-users to the decision tree, following the assessment of the expert opinions for the tree structure, we developed a web-based tool, which we called, StatXFinder. It was developed with PHP scripting language (version 5.3.10) and MySQL database (version 5.5.31) on Apache (version 2.2.4) Linux Server. The user interfaces were created using HTML, and the basic operations of test selection steps were coded in PHP. Furthermore, the user interface of StatXFinder was made as easy to use as possible with the addition of JavaScript and JQuery UI elements. The communication between the user interface of StatXFinder and MySQL database was performed with PHP scripts.

### User assessment of StatXFinder

In order to determine the usability and the user satisfaction of the web-based tool, 36 volunteer researchers were included to the study. The researchers had different academic titles and disciplines, and were applicants seeking consultation from Department of Biostatistics and Medical Informatics at Ege University, Faculty of Medicine, during a two- week period, between January 26, 2015 and February 9, 2015. The test recommendations in the decision tree were separated into two groups as tests recommended by answering more than five questions (Group 1) and tests recommended by answering five or less questions (Group 2), to be used in the assessment of the tool. Five different statistical test recommendations, from each group were randomly selected, and the groups were categorized as “difficult” and “easy” respectively. While Group 1 comprised of paired samples t-test, one sample t test, Kappa statistics, Pearson correlation and two-way ANOVA; Group 2 tests included Chi-square test, Spearman rank correlation, simple linear regression, Bartlett or Levene Test and ROC test. The “Help” menu of the IBM SPSS version 21.0 and the examples in Rosner’s textbook were utilized to prepare the example cases related to these tests (Rosner 2000; IBM SPSS 2012). Each case was prepared as easily understandable, short texts comprising of a single paragraph containing medical cases that could be easily solved with the help of the questions in the decision tree (see Additional file 1). Using these cases, the researchers who had applied for consulting, were asked to identify, randomly, two different numbers between 1 and 5. The cases from “difficult” and “easy” groups were selected with regard to these two numbers and the researchers were made to read these cases, and were asked to determine the statistical tests appropriate for the cases using the tool and the accuracy of the answers was calculated.

After the questions in the cases were answered via the tool, first the SUS (see Additional file 2), comprising of 10 questions, was applied to the participants to assess the usability of the tool and the SUS total score was calculated for each participant (Brooke 1996). The total score of the SUS takes a value between 0 and 100, and high total score indicates that the usability of the tool is high. In addition, the USQ (see Additional file 3) was applied to determine the user satisfaction of the tool. In this questionnaire, comprising of 14 questions, there are 7 open-ended questions and 7 multiple-choice questions. With the first 7 questions, in the first section of the questionnaire, the user information was obtained and with the remaining 7 questions in the last section, the users were requested to indicate their general satisfaction level for the tool, the features of the tool they liked and did not like, and their recommendations about the tool. For categorical variables such as academic title and sex, the frequency tables were generated and descriptive statistics were calculated for continuous variables such as age and SUS total score. Shapiro–Wilk test was used to examine whether the total score for each categorical variable was distributed normally or not. Mann–Whitney U test and Kruskal–Wallis test were used to compare the SUS score total results for the satisfaction questionnaire with regard to the user information obtained. Chi-square test was used to examine the relationship between the categorical variables in the user information. All analyses were conducted using the IBM SPSS software version 21.0 and SUS, USQ and the cases could also be downloaded in pdf format at the StatXFinder’s project website (IBM SPSS 2012).

**Fig. 1:**
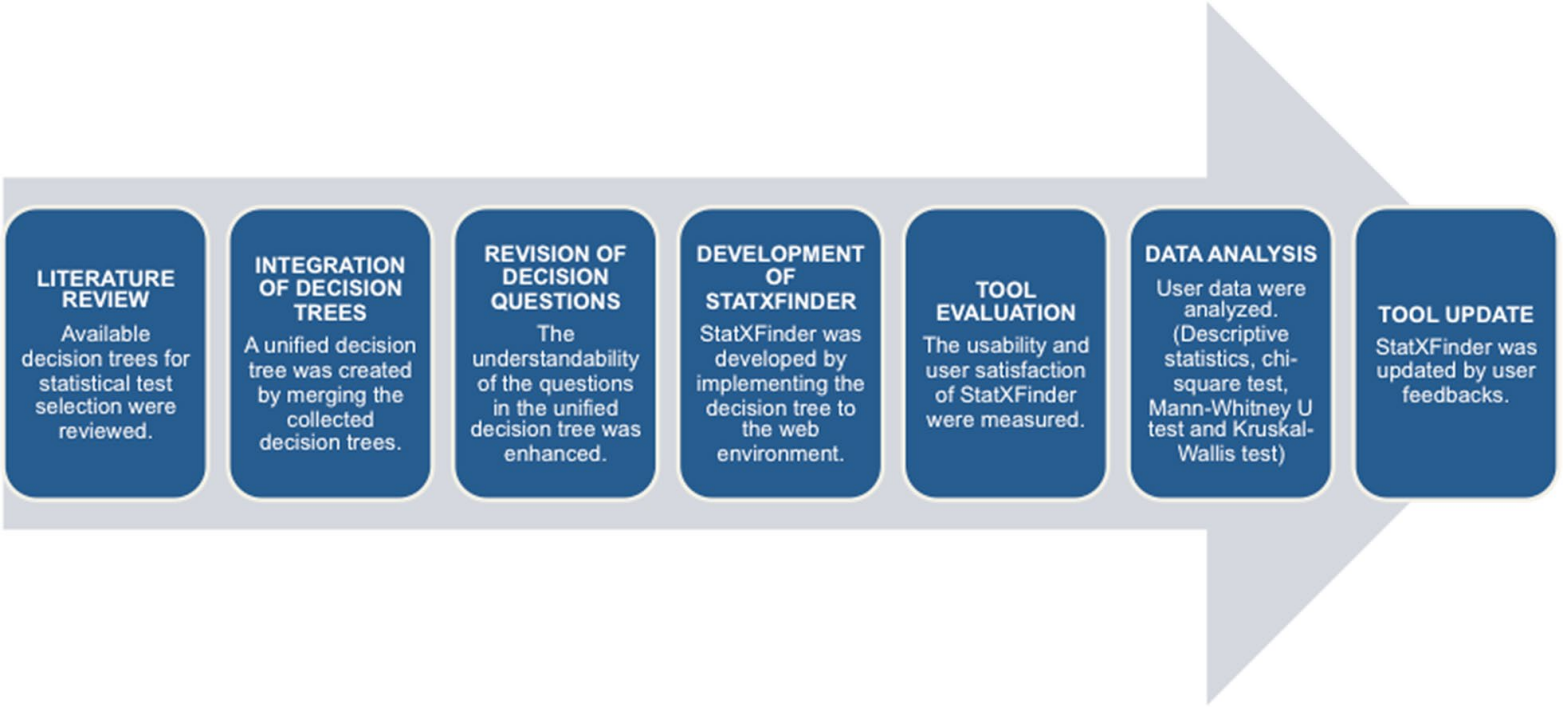
Schematic diagram of our study workflow

As a result of the user assessments, 15 questions and 8 test recommendations in the decision tree, constructed in the previous study were updated and 5 new questions and 8 new test recommendations were added to the decision tree (Suner et al. 2014). A schematic diagram on the workflow of our study is shown in Fig. 1. The tool, with the latest updates, provides users with recommendations for 85 statistical tests asking 44 different questions.

## Results

### Unified decision tree

After the manual online search, 10 different decision trees were determined and were included in the study. The decision trees used in the study and the corresponding tests were presented in detail in Table 1. When the tests guided by these decision trees were examined it was found that t test, ANOVA, Chi square test, Kruskal–Wallis H test, Mann–Whitney U test, and Wilcoxon signed rank test were the ones most frequently provided with decision support. Among the decision trees, the most comprehensive one in terms of the number of its corresponding statistical tests was Rosner’s tree structure. Accordingly, Rosner’s decision tree, acknowledged as the base decision tree, provided decision support in the selection of 36 statistical tests. However, Rosner’s tree structure did not provide decision support for test methods such as Mann–Whitney U test, Wilcoxon rank sum test, Dunn test, Bonferonni’s test, Tukey’s HSD test, Scheffe’s test, and Dunnett’s test, Sign test, Kendall W Test, Friedman test and Cochran’s Q test.

**Table 1:** Statistical test selection approaches included in the study after manual search, and their corresponding tests

Reference	No of tests	Suggested statistical tests
Jaykaran	25	One sample t test, unpaired t test, paired t test, one-way ANOVA, repeated measures ANOVA, Pearson correlation, simple linear regression, simple logistic regression, multiple linear regression, multiple nonlinear regression, multiple logistic regression, statistics for one group description (mean, standard deviation, median, interquartile range, proportion), Wilcoxon rank sum test, one sample binomial test with exact methods, Mann–Whitney U test, Fisher’s exact test, Wilcoxon signed rank test, McNemar’s test, Kruskal–Wallis H test, Chi-square test of independence, Friedman test, Cochrane Q test, Spearman’s correlation, contingency coefficients, nonparametric regression
Twycross and Shields	11	Unpaired (independent) t test, paired (dependent) t test, one-way ANOVA, repeated measures ANOVA, Mann–Whitney U test, Wilcoxon signed rank test, Kruskal–Wallis H test, Chi-square test of independence, Friedman test, Spearman’s correlation, Kendall’s coefficient of concordance
Gunawardena	14	Unpaired (independent) t test, paired (dependent) t test, one-way ANOVA, repeated measures ANOVA, Pearson correlation, multiple linear regression, Mann–Whitney U test, Wilcoxon signed rank test, McNemar’s test, Kruskal–Wallis H test, Chi-square test of independence, Friedman test, Spearman’s correlation, contingency coefficients
Marusteri and Bacarea	11	One sample t test, unpaired (independent) t test, paired (dependent) t test, one-way ANOVA, repeated measures ANOVA, Welch’s corrected unpaired t test, Wilcoxon rank sum test, Mann–Whitney U test, Wilcoxon signed rank test, Kruskal–Wallis H test, Friedman test
Gaddis and Gaddis	9	Mann–Whitney U test, Fisher’s exact test, Wilcoxon signed rank test, Chi-square test of independence, Kruskal–Wallis H test, Friedman test, Chi-square goodness of fit test, RxC (Rows by Columns) test, Kolmogorov-Simirnov test
Nayak and Hazra	30	Unpaired (independent) t test, paired (dependent) t test, one-way ANOVA, repeated measures ANOVA, Pearson correlation, Wilcoxon rank sum test, Mann–Whitney U test, Fisher’s exact test, Wilcoxon signed rank test, McNemar’s test, Kruskal–Wallis H test, Friedman test, Cochrane Q test, Spearman’s correlation, Kendall’s coefficient of concordance, RxC test, Tukey’s HSD Test, Newman-Keuls test, Bonferonni’s test, Dunnet’s test, Scheffe’s test, Dunn’s test, risk ratio, odds ratio, Chi-square test for trend, logistic regression, interclass correlation coefficient, Bland–Altman plot, Cohen’s Kappa statistics, Chi-square test for 2 × 2 table
McCrum-Gardner	13	Unpaired (independent) t test, One-way ANOVA, Repeated measures ANOVA, Mann–Whitney U test, Paired (dependent) t test, Wilcoxon signed rank test, McNemar’s test, Kruskal–Wallis H test, Friedman test, Cochrane Q test, RxC test, Chi-square test for 2 × 2 table, Chi square test for 2 × C table
UCLA	31	One sample t test, unpaired (independent) t test, paired (dependent) t test, one-way ANOVA, repeated measures ANOVA, Pearson correlation, simple linear regression, simple logistic regression, multiple linear regression, multiple logistic regression, repeated measures logistic regression, factorial ANOVA, ordered logistic regression, factorial logistic regression, one-way ANCOVA, one sample binomial test with exact methods, Mann–Whitney U test, Fisher’s exact test, Wilcoxon signed rank test, Kruskal–Wallis H test, Chi-square test of independence, Friedman test, one-sample median, Chi-square goodness of fit test, McNemar’s test, Spearman’s correlation, one-way MANOVA, multivariate multiple linear regression, factor analysis, canonical correlation, discriminant analysis
Mertler	17	Pearson correlation, simple linear regression, multiple linear regression, path analysis, unpaired (independent) t test, one-way ANOVA, one-way ANCOVA, factorial ANOVA, factorial ANCOVA, one-way MANOVA, one-way MANCOVA, factorial MANOVA, factorial MANCOVA, simple logistic regression, discriminant analysis, factor analysis, principal components analysis
Rosner	36	One sample t test, One sample binomial test with exact methods, Paired (dependent) t test, Unpaired (independent) t test, One-way ANOVA, Pearson correlation, Simple linear regression, Multiple linear regression, Multiple logistic regression, Welch’s corrected unpaired t test, One-way ANCOVA, One sample z-test, One sample binomial test with normal theory methods, One sample Poisson test, Two-sample F test to compare variances, Nonparametric methods for two-sample problem, Two-way ANOVA, Two-way ANCOVA, Higher-way ANOVA, Higher-way ANCOVA, Two sample test for comparison of incidence rates, One-sample test for incidence rates, Test of trend for incidence rates, Fisher’s exact test, McNemar’s test, Kruskal–Wallis H test, Spearman’s correlation, Cohen’s Kappa statistics, Chi-square test for 2 × 2 table, Chi-square test for 2 × C table, Nonparametric methods for one-sample problem, Nonparametric methods for more than two samples problem, Chi-square test for trends, Log-rank test, Cox proportional hazards model, Chi-square test for heterogeneity for R × C tables

A comprehensive and unified decision tree facilitating the selection of 85 statistical tests with 59 distinct recommendations was obtained by integrating the other 9 decision trees included in the study into Rosner’s tree structure, acknowledged as the base decision tree (see Additional file 4). Our unified decision tree comprises of 44 different decision questions, all of which are in the form of yes–no questions. During the manual examination and reorganization of the questions, items that could not be answered as yes–no such as, “number of ways in which the categorical variable can be classified”, were rewritten as, “Is there only one categorical variable which affects the continuous variable?” In addition, after determining and reorganizing questions which were not understandable, or would cause doubt for potential users of the decision tree, a question which was directed to the user as, “Only one variable of interest?”, was rewritten as, “Does your data set have only one variable?”, and added into the decision tree. In this way, 29 questions were rewritten and updated.

In the study, to render the questions more understandable, additional explanations were written for 44 questions. For example, a question such as “Does your data set have only one variable?” was added the explanation “Do you have only one variable to analyze such as age or sex? (For instance, do you want to compare the mean value of a continuous random variable such as age with a known population mean?)” and thus the questions were simplified enough for non-statistician users. Furthermore, a mini-glossary comprising of 44 terms was created by preparing explanations for the statistical terms in some of the questions. For instance, the term “variable” at one of the decision steps was defined as, “A characteristic that consists of two or more categories or values, and that differs from subject to subject or from time to time. Categories such as occupation or nationality, or values such as age or intelligence score are examples of variable. The opposite of variable is constant. The term ‘variable’ is often used as a shortened form of ‘random variable’”. All user questions in the decision tree and their corresponding explanations could be downloaded in pdf format at the StatXFinder’s website (see Additional file 5).

Each branch of the decision tree, which was modified with the updated questions and newly formed question explanations was then assessed under the guidance of five expert statisticians. While reorganizing the order of the questions for the users at the decision steps, in accordance with the opinions of the experts, it was approved that all recommendations met the requirements of the questions. Accordingly, it was possible to acquire the recommendations for ordered logistic regression with a minimum of four questions and the recommendation for the Chi-square test for trend with a maximum of 11 questions. When the questions were examined in terms of their content, it was found that they comprised of “number of variables”, “number of samples”, “dependency of samples”, “normality assumption”, “scale type of variables”, and additional types of questions for some specific cases.

**Fig. 2:**
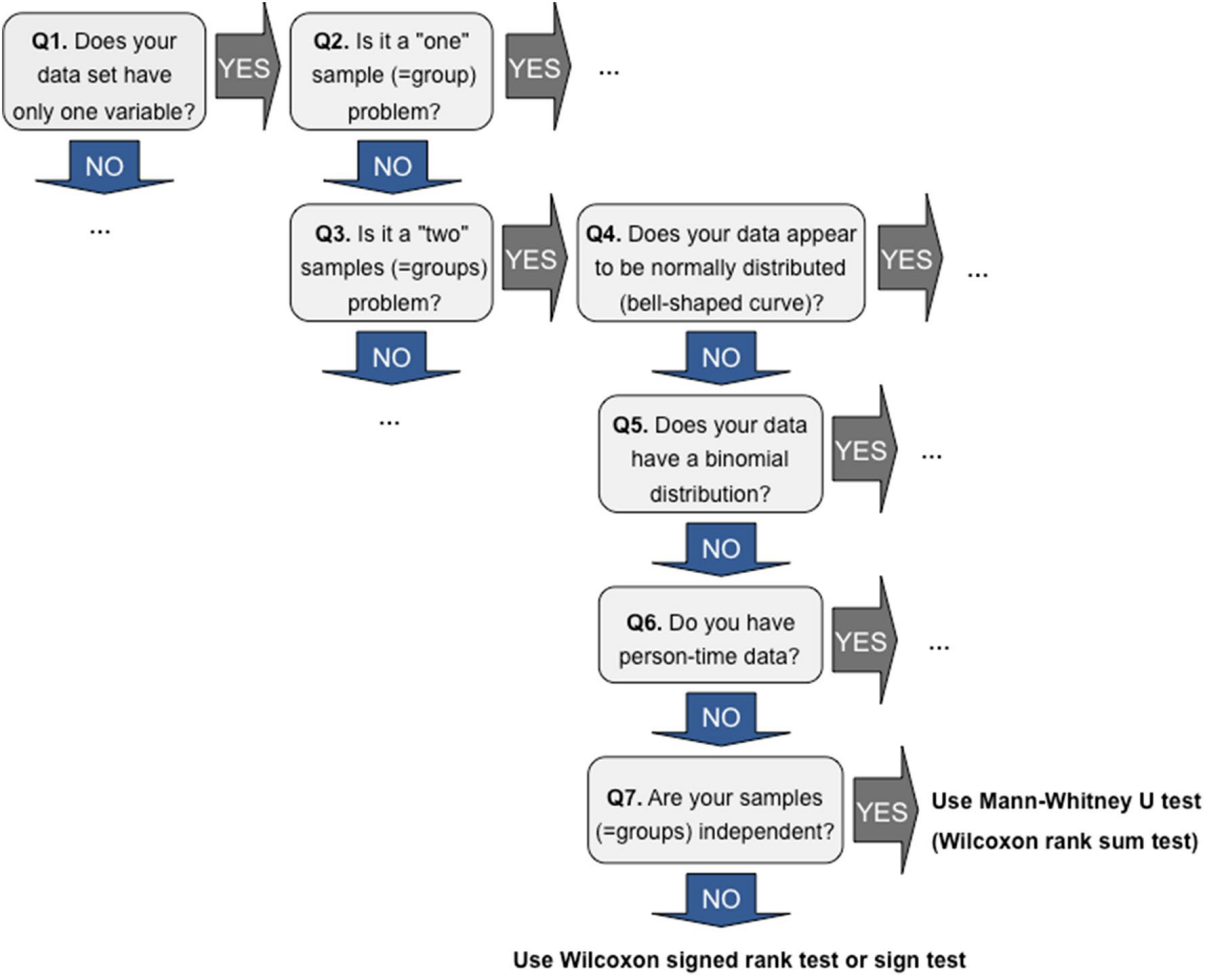
An example of a decision tree for appropriate statistical test selection

An example of a decision process pertaining to the decision tree developed here is presented in Fig. 2. In this example decision processes for the selection of Mann–Whitney U Test (Wilcoxon rank sum test) and Wilcoxon signed rank test or sign test were illustrated. If the data set of the user had only one variable and the user wished to test not normally distributed two samples, the appropriate test could be selected considering independence of the samples. In addition, the decision tree guides the user in regard to how the normality assumption of the data set could be tested.

### The usage of StatXFinder

**Fig. 3:**
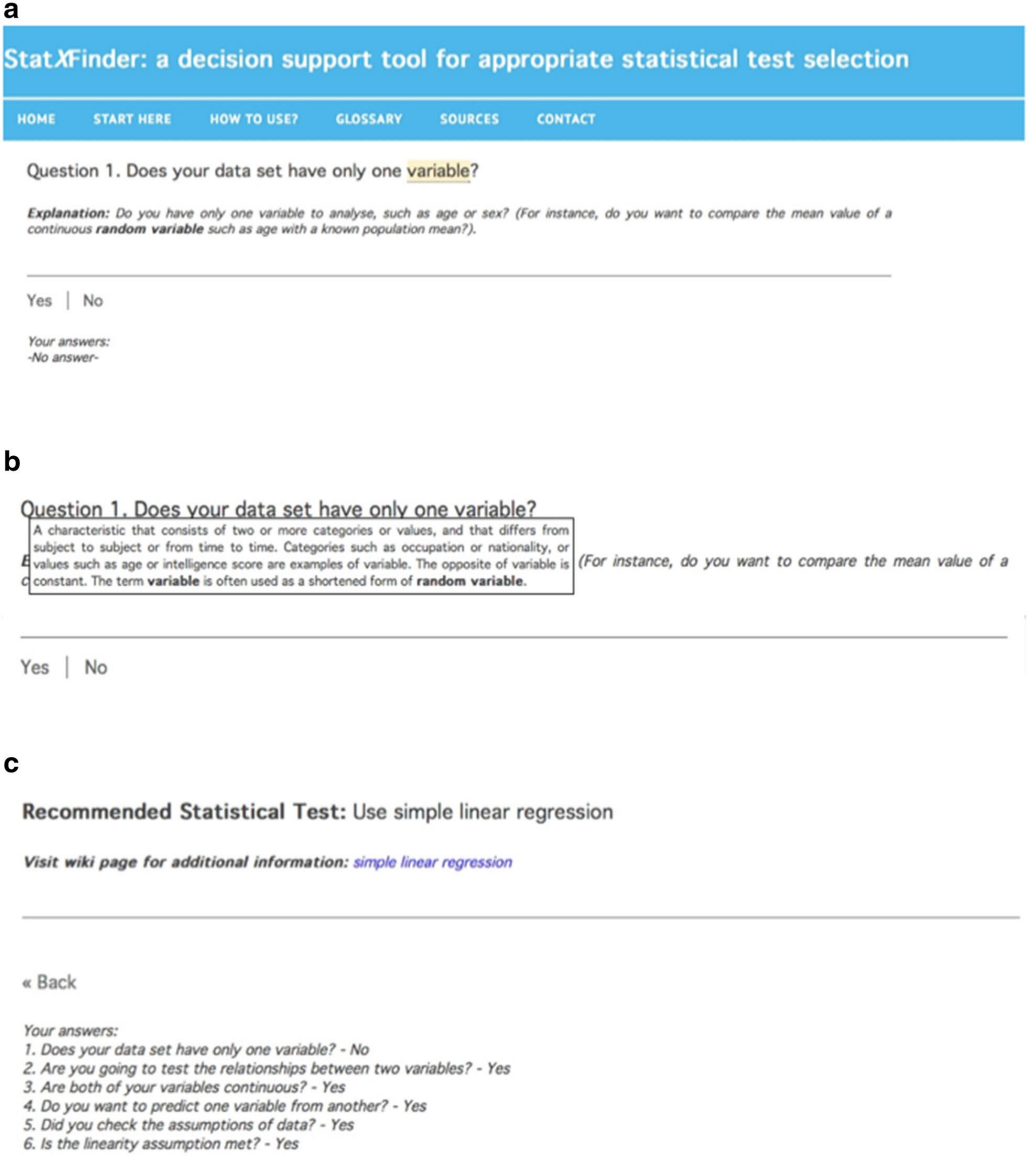
The screenshots of StatXFinder. **a** StatXFinder optionally provides explanations for each question. **b** If there is any statistical term in the question, the explanation of that term can be viewed by moving the *cursor* over that term. **c** By giving the required answers at each decision step, StatXFinder ends the decision process by offering a recommendation

In the last stage of the study, the potential users of the decision tree were provided with access to StatXFinder. Figure 3a shows the first decision question and the user interface regarding appropriate statistical test selection. The user provides yes–no answers to the questions shown by the interface in order to proceed to next step. By answering all the questions, the tool provides recommendations in accordance with the requirements defined by the user. Optional question explanations were provided at the decision steps, and the selection of the answer appropriate for the user’s data set was facilitated. If the user wished to change the answer given, they could correct the answer with the “back” button on the screen, and previous answers can be seen at any step. In addition, the statistical terms in the questions were highlighted and the tool allows for the user to view the explanation of the term by moving the mouse pointer over that term (Fig. 3b). Also, the explanations prepared for statistical terms are presented in the glossary section on the website.

As an example for the use of StatXFinder, let us assume that a researcher is trying to determine if the use of an oral contraceptive caused an increase in systolic blood pressure in females. Moreover, let us presume that the researcher measured the systolic blood pressure (mm/hg) values for 15 females between ages 20 and 39, before and after drug use. In addition to this, let us assume that the difference of the systolic blood pressure values measured before and after the drug use is not normally distributed. In this case, the groups are dependent to each other since pair wise observations were conducted for the same individuals before and after drug use. In this case, the researcher would obtain the decision on which test or tests he/she could use in analyzing the data using StatXFinder following the decision steps presented in Table 2. The decision steps of this example were also illustrated in Fig. 2.

**Table 2:** The questions directed in a particular order for an example hypothesis test

Step	Questions	The statistical terms observed in the questions*	Definitions	Answers
1	Does your data set have only one variable?	Variable^a^	Since the example only addresses the values that a single variable, in the form of systolic blood pressure (mm/hg), could take, the answer to this question should be “yes”	Yes
2	Is it a “one sample” problem?	Sample^b^	Since the experimenter obtained measurement values from the patient before and after administering drugs, there are two groups of data in question	No
3	Is it a “two samples” problem?	Sample^b^	The experimenter has two groups of data such as pre and post administering	Yes
4	Does your data appear to be normally distributed (bell-shaped curve)?	Normally distributed^c^	The answer is given as “no” since the analysis was conducted with the assumption that the measurement values were not normally distributed and the sample size is smaller than 30	No
5	Does your data have a binomial distribution?	Binomial distribution^d^	Since systolic blood pressure (values) do not have two possible outcomes as “success” and “failure”, it does not show a binomial distribution and the answer for this question should be “no”	No
6	Do you have person-time data?	Person-time data^e^	Since the blood pressure measurement value is not a variable which is observed over time such as some individuals who developed lung cancer over a year (time) this question was answered as “no”	No
7	Are your samples (=groups) independent?	Sample^b^, independent^f^	Since two measurements were conducted on the same group before and after administering drugs to the patients, such as pre-treatment vs. post-treatment, the systolic blood pressure values measured in these two groups were dependent to each other; therefore, the question was answered as no	No
Recommendation—use Wilcoxon signed rank test or Sign test				N/A

^a^A characteristic that consists of two or more categories or values, and that differs from subject to subject or from time to time. Categories such as occupation or nationality, or values such as age or intelligence score are examples of variable. The opposite of variable is constant. The term variable is often used as a shortened form of random variable

^b^A subset of cases drawn or selected, according to some specified criteria, from a larger set or population of cases with the purpose of estimating characteristics of the larger set or population, drawing inferences about the these characteristics and generalizing results from sample to population. A sample should be representative of the population from which it is drawn in order to be useful. For instance, to find out the relationship between drug abuse and mental health, it would be possible or practical to investigate this relationship by taking a sample of the population. Thus, it would be possible to determine to what extent this relationship is likely to be found in the population

^c^A theoretical distribution which shows the frequency or probability of all the possible values that a continuous variable can take. This distribution is bell shaped. The horizontal axis of the distribution represents all possible values of the variable while the vertical axis represents the frequency or probability of those values. In any normal distribution: (1) 68 % of the observations fall within σ of the mean μ, (2) 95 % of the observations fall within 2σ of μ, and (3) 99.7 % of the observations fall within 3σ of μ. This is known as 68–95–99.7 rule. It is also called the gaussian distribution

^d^The probability distribution of the number of successes in n independent Bernoulli trials, such as a person passing or failing or being a woman or a man, where each trial has two outcomes (conveniently labeled success and failure), and the probability of success p is the same for each trial

^e^Data referring to a measurement obtained by combining person data and time data. It is obtained as the sum of individual units of time that the subjects in the study population have been exposed to certain risk. It can also be obtained as the number of persons at risk of the event of interest multiplied by the average length of the study period

^f^Independence is a characteristic of observations or random events. Essentially, the term is used to describe the property of independence of events or sample observations. It is an assumption required by many statistical tests. Independent variable is the variable in an experiment that is under the control of, and may be manipulated by, the experimenter. In regression analysis it is the variable being used to regress or predict the value of the dependent variable. It is also commonly known as regressor, predictor, or explanatory variable

### Usability and user satisfaction of StatXFinder

The accuracy rate of the statistical test recommendations obtained by the users, with the cases applied, were 83.3 % for “difficult” tests, and 88.9 % for “easy” tests. The total System Usability Score (SUS) score was calculated with regard to user’s level of agreement with the expressions in the SUS, and the mean score of the tool for 36 people was found 87.43 ± 10.01 (minimum: 70—maximum: 100). The SUS scores to be expressed in a more detailed fashion in Table 3. Li et al. asserted that there was no consensus on the limits of acceptability for the SUS scores; however scores below 70 are evaluated as ‘passable’, values between 70 and 80 s are accepted as ‘good products’, and score values 90 and above are acknowledged as ‘superior’ (Li et al. 2013). The SUS score of StatXFinder was quite high and this score may be evaluated as good product.

**Table 3:** The usability testing results of StatXFinder

System Usability Scale items	Mean ± SD (n = 36)
1. I think that I would like to use the StatXFinder frequently	4.30 ± 0.86
2. I found the StatXFinder unnecessarily complex	1.44 ± 0.94
3. I thought the StatXFinder was easy to use	4.72 ± 0.57
4. I think that I would need the support of a technical person to be able to use the StatXFinder	1.61 ± 1.10
5. I found the various functions in the StatXFinder were well integrated	4.25 ± 0.99
6. I thought there was too much inconsistency in the StatXFinder	1.25 ± 0.86
7. I would imagine that most people would learn to use the StatXFinder very quickly	4.39 ± 0.80
8. I found the StatXFinder very cumbersome to use	1.19 ± 0.47
9. I felt very confident using the StatXFinder	4.61 ± 0.55
10. I needed to learn a lot of things before I could get going with the StatXFinder	1.81 ± 0.92
Total System Usability Scale score (0–100; Higher score means more user friendly tool)	87.43 ± 10.01

*SD* standard deviation

The personal information obtained from the first section of the User Satisfaction Questionnaire (USQ) and the information about the users’ skills are summarized in Table 4. These data showed that the participants were generally comprised of women (61.1 %) and had the academic title of “Master of Science (MSc) Student” (44.4 %). The mean age of the participants was 33.47 ± 9.83 (minimum: 23—maximum: 58). The levels of computer skills of the participants were generally “advanced” (72.2 %), their levels of English proficiency were “advanced” (61.1 %), and their levels of statistical knowledge were “average” (44.4 %). Eight of the participants (22.2 %) had never conducted a statistical analysis using a statistical software package. The total SUS score for each categorical variable was not normally distributed (p value <0.05). A statistically significant difference could not be seen between total system usability score and academic title, gender, conducting statistical analysis with a statistical software package, level of computer use skills, level of English proficiency, level of statistics knowledge, recommending the tool to others, and the level of appreciation of the tool (p value >0.05). When Table 4 is examined, although it is seen that the SUS total score increased as the level of computer skills, level of English proficiency and the level of statistical knowledge, this increase was not found statistically significant (p value >0.05). While evaluating the tool as a whole, nine (52.9 %) participants who had the academic title of “Master of Science (MSc) Student”, five (29.4 %) participants with the “Doctor of Philosophy (PhD)” academic title, two (11.8 %) participants with “MSc” academic title, and one (5.9 %) participant with “Medical Doctor (MD)” academic title, marked the “I like it very much” option in the User Satisfaction Questionnaire.

**Table 4:** The frequency table and descriptive statistics of total SUS score for users’ attributes

Question	Answer	Frequency (%)	Descriptive Statistics for total SUS score (Mean ± SD), (Min–Max)	P-value
Academic title	MSc. Student	16 (44.4 %)	88.28 ± 11.09 (70–100)	0.766^a^
	MSc.	2 (5.6 %)	90 ± 0 (90–90)	
	PhD. Student	2 (5.6 %)	90 ± 0 (90–90)	
	PhD	11 (30.6 %)	87.95 ± 9.34 (72.5–97.5)	
	MD	5 (13.9 %)	81.50 ± 11.94 (70–100)	
Gender	Female	22 (61.1 %)	86.82 ± 11.11 (70–100)	0.961^b^
	Male	14 (38.9 %)	88.39 ± 8.29 (70–97.5)	
Age	Mean ± SD (Min–Max)	33.47 ± 9.83 (23–58)	–	–
Level of computer use skills	Expert	7 (19.4 %)	90.71 ± 10.10 (75–100)	0.530^a^
	Advanced	26 (72.2 %)	87.02 ± 10.61 (70–100)	
	Average	3 (8.3 %)	83.33 ± 8.29 (70–97.5)	
	Elementary	–	–	
	Beginner	–	–	
Level of english proficiency	Proficient	6 (16.7 %)	95.71 ± 3.16 (92.5–100)	0.110^a^
	Advanced	22 (61.1 %)	85.45 ± 10.11 (72.5–100)	
	Intermediate	8 (22.2 %)	87.19 ± 11.26 (70–97.5)	
	Elementary	–	–	
	Beginner	–	–	
Level of statistics knowledge	Expert	3 (8.3 %)	90.83 ± 1.44 (90–92.5)	0.077^a^
	Advanced	11 (30.6 %)	92.50 ± 8.94 (75–100)	
	Average	16 (44.4 %)	82.81 ± 10.64 (70–100)	
	Elementary	6 (16.7 %)	88.75 ± 8.18 (72.5–95)	
	Beginner	–	–	
Using a statistical software package	Yes	28 (77.8 %)	87.41 ± 10.01 (70–100)	0.924^b^
	No	8 (22.2 %)	87.5 ± 10.69 (70–100)	

*SD* standard deviation

^a^Kruskal–Wallis test

^b^Mann–Whitney U test

According to USQ results, the most appreciated features of StatXFinder were indicated as, “the understandability of the questions with the help of the explanations, practicability, ease to use, step by step progression, presenting the previous answers, being time-saving, accessibility over the Internet, glossary support, and correctability of the answers”. The participants did not appreciate the tool’s language being English. None of the participants indicated a feature to be removed from the tool. The participants recommended that multivariate analysis methods be added to the tool, brief information about the tests recommended be provided, and references to these tests be given. Thirty-five of the participants (97.2 %) indicated that they would recommend the tool to others. When the general level appreciation of the tool is assessed, none of the participants marked the, “I did not like at all” option, while 17 (47.2 %) marked “I like it very much”, 18 (50 %) marked “I like it”, and 1 (2.8 %) marked “No idea”. When the results pertaining to the level of appreciation obtained from the cross-tables are examined, a statistically significant relation could not be found between the categorical variables. As a result of the Chi-square test 76 % (13 participants) of the participants who indicated that they had liked the tool very much had an “advanced” level of computer skills, 47.1 % (8 participants) had an “advanced” level of English proficiency, and 52.9 % (9 participants) had an “advanced” level of statistics knowledge (p value >0.05). 50 % (3 participants) of the participants with a “beginner” level of statistics knowledge marked the “I like it” option, while the other 50 % (3 participants) marked the “I like it very much” option. According to the results of the statistical analysis, 75 % (12 participants) of the participants who indicated their levels of statistics knowledge as “average” marked the “I like it” option, 18.8 % (3 participants) of them marked the “I like it very much”, and 6.3 % of them (1 participant) marked the “No idea” option. 51.4 % (18 participants) of the participants who indicated that they would recommend the tool to others marked the “I like it” option, while 48.6 % (18 participants) marked the “I like it very much”.

### Discussion and conclusions

An accurate interpretation of biomedical research findings is only possible with the determination and implementation of statistical analysis methods appropriate for the data collected in the research processes (du Prel et al. 2010; Charan and Saxena 2012). On the other hand, a data analysis with an inaccurate test selection leads to false inferences (Nyirongo et al. 2008). Therefore, the selection of statistical test or tests in the transformation of the data collected in a biomedical study into knowledge is crucial to researchers in testing his/her hypotheses or accurately reading the findings he/she obtained.

Although currently there are various decision trees for selection of statistical tests appropriate for the use of researchers, it is seen that when these tree structures are assessed separately, they offer decision support for a limited number of tests. This requires the researchers to switch between present decision trees and to select the one most appropriate for their purpose and therefore complicates their practical use. Moreover, the complexity of some questions may lead non-statisticians to incorrect tests (Jaykaran 2010; Nayak and Hazra 2011).

In this study we devised a unified tree structure for the selection of more statistical tests by piecing together the decision trees, and we enriched our decision tree by examples and explanations that would clarify the concepts, which could be difficult to understand. We also developed a user-friendly tool, StatXFinder, and we provided potential users with the access to the decision tree in the web environment.

When the decision trees included in the study were assessed in terms of the number of their corresponding statistical test methods, it was found that the decision tree developed by Rosner was the most comprehensive one (Rosner 2000). Also, Rosner’s tree structure differed from others in terms of ease-of-use, with its relatively simpler questions in yes–no question form. Therefore, Rosner’s tree formed the backbone of the decision tree we devised in the study. However, Rosner’s decision tree differed from others in that it did not include commonly used multiple comparison tests and nonparametric statistical tests. Thus, we integrated the decision processes of the multiple comparison and nonparametric statistical tests determined in others into Rosner’s decision tree and devised a more comprehensive one which could address the needs of a wider variety of scientific domains.

While StatXFinder can offer recommendations for survival analysis, parametric and nonparametric tests, it also includes some other statistical tests used frequently in medical domain such as multiple comparison tests and ROC analysis. However, StatXFinder could not provide, decision support for multivariate statistical test methods, for now; but it is possible to add these tests into StatXFinder which has a modular and extendable structure, and in the near future we plan to add different statistical tests into StatXFinder. Different from most of the statistical test selection decision trees, Wh-questions in the non-polar form were avoided for limiting the alternatives that could be selected by the users; instead the users were allowed to make a binary selection with polar yes–no questions. Thus, the users are expected to determine whether the requirements in the questions were met instead of giving relatively complex answers to the questions as in other decision trees. Even though yes–no answers are given to simplified questions, the appropriateness of the tests recommended by StatXFinder to the data set completely depends on the correct answers given to the questions at the decision steps as a nature of decision support systems.

When the results obtained from the user assessment are examined, it is seen that the accuracy rate of the tool is rather high. This indicates that the users can select the correct answers since questions directed by the tool are easily understandable. With the improvements made after user recommendations, the usability of the tool increased. When the results of the SUS scores are examined, it can be said that the usability of the tool is rather high. The lack of difference between the SUS scores and the satisfaction levels of participants with different academic titles and knowledge levels indicates that the tool can be used easily by anyone in the biomedical domain who needs to conduct statistical analysis. When the opinions obtained from the USQ are examined, it can be said that the level of appreciation of the tool is quite high, and almost all of the participants thought of recommending the tool to others.

The opinions obtained from the participants indicate that features such as online access to the tool, time saving in test selection, understandability and practicability, listing the answers given, ease-of-use due to step-by-step progression, glossary support, and allowing for correcting the answers given were appreciated. Since Turkish language support was demanded by the participants, the tool is planned to be constructed for various languages. The links containing explanatory information about the recommended statistical tests were inserted to the tool in line with the recommendations by the participants.

In conclusion, we developed a web-based tool called StatXFinder in order to allow biomedical researchers with limited statistics knowledge to make decisions in selecting appropriate statistical tests. StatXFinder, which was developed by integrating present decision trees and updating the question structures and content, is open to development and the addition of new tests with its modular structure. StatXFinder is freely available at http://webb.deu.edu.tr/tb/statxfinder.

## Additional files

10.1186/s40064-015-1421-9 Example cases for StatXFinder usage.
10.1186/s40064-015-1421-9 System usability score questionnaire.
10.1186/s40064-015-1421-9 User satisfaction questionnaire.
10.1186/s40064-015-1421-9  The list of recommended statistical tests by StatXFinder.
10.1186/s40064-015-1421-9  Decision questions and corresponding explanations.

## Abbreviations

SOFA: Statistics Open For All
SUS: System Usability Scale
USQ: User Satisfaction Questionnaire
MSc: Master of Science
PhD: Doctor of Philosophy
MD: Medical Doctor
RxC: Rows by Columns

## References

[CR1] Bettany-Saltikov J, Whittaker VJ (2014). Selecting the most appropriate inferential statistical test for your quantitative research study. J Clin Nurs.

[CR2] Brooke J (1996). SUS-A quick and dirty usability scale. Usability Eval Ind.

[CR3] Charan J, Saxena D (2012). Suggested statistical reporting guidelines for clinical trials data. Indian J Psychol Med.

[CR4] Corp I. B. M. (2012) IBM SPSS statistics for Windows, version 21.0, Armonk, NY, USA

[CR5] Cramer D, Howitt DL (2004). The Sage dictionary of statistics: a practical resource for students in the social sciences.

[CR6] Du Prel J-B, Röhrig B, Hommel G, Blettner M (2010). Choosing statistical tests: part 12 of a series on evaluation of scientific publications. Dtsch Arztebl Int.

[CR7] Everitt BS, Skrondal A (2010). The Cambridge Dictionary of Statistics.

[CR8] Gaddis GM, Gaddis ML (1990). Introduction to biostatistics: Part 5, statistical inference techniques for hypothesis testing with nonparametric data. Ann Emerg Med.

[CR9] Gunawardena N (2011). Choosing the correct statistical test in research. Sri Lanka J Child Health.

[CR10] Harris AHS, Reeder R, Hyun JK (2009). Common statistical and research design problems in manuscripts submitted to high-impact psychiatry journals: what editors and reviewers want authors to know. J Psychiatr Res.

[CR11] Jaykaran (2010). How to select appropriate statistical test?. J Pharm Negat Results.

[CR12] Johnson LR, Karunakaran UD (2014). How to Choose the Appropriate Statistical Test Using the Free Program “Statistics Open For All” (SOFA). Ann Community Heal.

[CR13] Lang T (2004). Twenty statistical errors even you can find in biomedical research articles. Croat Med J.

[CR14] Leeper JD (2006) What statistical analysis should I use? http://www.ats.ucla.edu/stat/mult_pkg/whatstat/. Accessed 12 Jun 2015

[CR15] Li LC, Adam PM, Townsend AF (2013). Usability testing of ANSWER: a web-based methotrexate decision aid for patients with rheumatoid arthritis. BMC Med Inform Decis Mak.

[CR16] Marusteri M, Bacarea V (2010). Comparing groups for statistical differences: how to choose the right statistical test?. Biochem Medica.

[CR17] McCrum-Gardner E (2008). Which is the correct statistical test to use?. Br J Oral Maxillofac Surg.

[CR18] Mertler CA, Vannatta RA (2002). Advanced and multivariate statistical methods.

[CR19] Nayak BK, Hazra A (2011). How to choose the right statistical test?. Indian J Ophthalmol.

[CR20] Normando D, Tjäderhane L, Quintão CCA (2010). A PowerPoint-based guide to assist in choosing the suitable statistical test. Dental Press J Orthod.

[CR21] Nyirongo V, Mukaka M, Kalilani-Phiri L (2008). Statistical pitfalls in medical research. Malawi Med J.

[CR22] Okeh U (2009). Statistical problems in medical research. East Afr J Public Health.

[CR23] Rosner B (2000). Fundamentals of biostatistics.

[CR24] Sahai H, Khurshid A (2002). Pocket dictionary of statistics.

[CR25] Strasak AM, Zaman Q, Pfeiffer KP (2007). Statistical errors in medical research—a review of common pitfalls. Swiss Med Wkly.

[CR26] Suner A, Karakülah G, Dicle O (2014). Towards a web-based decision support tool for selecting appropriate statistical test in medical and biological sciences. Stud Health Technol Inform.

[CR27] Team RDC (2008) R: A Language and Environment for Statistical Computing. R Found. Stat. Comput. 1:ISBN 3–900051–07–0

[CR28] Twycross A, Shields L (2004). Statistics made simple. Part 4. Choosing the right statistical test. Paediatr Nurs.

[CR29] Wiles S, Bishop AL (2013) Common mistakes in data presentation and statistical analysis: how can the BioStat Decision Tool help? In: PeerJ Prepr. https://peerj.com/preprints/92/. Accessed 12 Jun 2015

