# Supplementary material for: StatXFinder: a web-based self-directed tool that provides appropriate statistical test selection for biomedical researchers in their scientific studies
Source: Springerplus. 2015 Oct 22;4:633. doi: 10.1186/s40064-015-1421-9 (PMC4627976; doi:10.1186/s40064-015-1421-9)
Supplement: Supplementary file 1 — 10.1186/s40064-015-1421-9 Example cases for StatXFinder usage. [file 40064_2015_1421_MOESM1_ESM.pdf]

EXAMPLE CASES FOR STATXFINDER USAGE

1. The blood pressure values of 50 male patients, between the ages of 35 and 44, who had been selected for a study, were measured as mmHg. After some treatment, the blood pressure values were measured again, and it was seen that the blood pressure values obtained from both measurements were distributed as a bell-shaped curve, i.e. exhibited a normal distribution. Which statistical method would you use in determining whether there is a statistically significant difference between the mean blood pressure values measured before and after the treatment?

   Answer= Paired Samples t-test

   (Number of Steps: 6 ➔ Yes-No-Yes-Yes-Yes-No)

2. A researcher wishes to test whether the mean IQ scores of 35 students are different from the mean IQ score value of 100, which had been obtained in the previous studies. If the IQ scores of the students are known to be distributed as a bell-shaped curve, i.e. exhibit a normal distribution, which statistical method would you use to determine whether the mean of the selected sample group is different from the mean value obtained in the previous studies?

   Answer= One Sample t test

   (Number of Steps: 6 ➔ Yes-Yes-Yes-Yes-Yes-No)

3. 2 different questionnaires querying daily diets were sent to the nurses in different regions of the county. In the questionnaires, they were asked how much they consumed 100 different foods and the weekly meat consumption amounts were obtained. The weekly meat consumption amounts were evaluated as less than 1 serving per week and 1 and more servings per week, with regard to the answers obtained. How can the reproducibility of these two different questionnaires be measured?

   Answer= Kappa Statistics

   (Number of Steps: 6 ➔ No-Yes-No-No-No-No)

4. In a study for determining whether environmental factors cause a rise in serum cholesterol level, which is an important risk factor for the etiology of the cardiovascular diseases, it is seen that the serum cholesterol levels obtained, from 100 genetically unrelated married couples, distributed as bell-shaped curve, i.e. exhibit a normal distribution. Which statistical method would you use to investigate the relationship between the serum cholesterol levels of husbands and wives?

   Answer= Pearson correlation

   (Number of Steps: 6 ➔No-Yes-Yes-No-No-Yes)

5. In a study investigating the effect of diet type with regard to sex on systolic blood pressure, the diet type is categorized into three (strict vegetarian, lacto-vegetarian and normal). It is known that the mean systolic blood pressure values for different diet types with regard to sex are distributed as bell-shaped curve, i.e. exhibit normal distributed, and the variablity of the data is similar. With regard to this information, which statistical method would you use to determine whether there is a difference between mean systolic blood pressure values for different diet types with regard to sex?

   Answer= Two-Way ANOVA

   (Number of Steps: 9 ➔ No-Yes-No-Yes-No-No-Yes-No-Yes)

6. Data regarding males living in the city, between the ages of 50 and 54, were examined retrospectively; and the ones who had died in the last month were selected from the data set. The causes of deaths were categorized as cardiovascular diseases and other diseases. The diets of the ones who had died due to cardiovascular diseases were categorized into three classes as high salt users, normal salt users and low salt users. Considering this information, which statistical method would you use to investigate the possible relationship between cause of death and high salt intake?

   Answer= Chi-square test

   (Number of Steps: 5 ➔ No-Yes-No-No-Yes)

7. For the calculation of the Apgar score, which is based on the physical states of the newborns measured at the 1st and the 5th minutes after birth, the score value is obtained by the sum of 5 components. Each component can take the values 0, 1 or 2 and a total score between 0 and 10 is obtained. Which statistical method would you use to investigate the relationship between the Apgar score values measured at the 1st and 5th minutes, which are not distributed as bell-shaped curve, i.e. do not exhibit a normal distribution?

   Answer= Spearman rank correlation

   (Number of Steps: 5 ➔ No-Yes-No-No-Yes)

8. In a study investigating whether there is any relationship between the estriol levels of pregnant women and newborn birth weight, the estriol levels are measured from the blood samples taken from pregnant women. Later, the newborn birth weights are obtained. Which statistical method would you use to investigate whether it is possible to predict newborn birth weights with regard to estriol levels?

   Answer= Simple linear regression

   (Number of Steps: 4 ➔ No-Yes-Yes-Yes)

9. In a study which investigates whether passive smoking have a measurable effect with regard to pulmonary health, pulmonary functions were measured for 6 different smoking states. These are non-smokers, passive smokers, non-inhaling smokers, light smokers (1 to 10 per day), moderate smokers (11 to 39 per day) and heavy smokers (40 and over per day). The measurements of pulmonary functions were performed by forced mid-expiratory flow (FEF). Which statistical method would you use to compare whether the variations of the data with regard to each smoking categories are similar?

   Answer= Bartlett Test or Levene Test

   (Numbers of Steps: 4 ➔ Yes-No-No-No)

10. A radiologist was asked to evaluate 109 computed tomographic scans. Some of these scans included a neurologic pathology. The real disease status of each sample was determined with reliable methods. The radiologist was asked to rate the evaluation results between 1 and 5 (1= definitely normal, 2= probably normal, 3= uncertain, 4= probably abnormal, 5= definitely abnormal). Which statistical method would you use to measure the diagnostic accuracy of this test?

    Answer= ROC Analysis

    (Number of Steps: 5 ➔ No-Yes-No-Yes-Yes)

NOTE: The **"**Help" Menu of the "IBM SPSS V. 21.0" software, and the examples of "Rosner, B. (2010). Fundamentals of Biostatistics. 5th Edition. Duxbury Press: USA " were used for preparing the sample scenarios.
