# Supplementary material for: StatXFinder: a web-based self-directed tool that provides appropriate statistical test selection for biomedical researchers in their scientific studies
Source: Springerplus. 2015 Oct 22;4:633. doi: 10.1186/s40064-015-1421-9 (PMC4627976; doi:10.1186/s40064-015-1421-9)
Supplement: Supplementary file 2 — 10.1186/s40064-015-1421-9 System usability score questionnaire. [file 40064_2015_1421_MOESM2_ESM.pdf]

Questionnaire No:

SYSTEM USABILITY SCALE

	Strongly disagree				Strongly agree
	1	2	3	4	5
1. I think that I would like to use this system frequently.					
2. I found the system unnecessarily complex.					
3. I thought the system was easy to use.					
4. I think that I would need the support of a technical person to be able to use this system.					
5. I found the various functions in this system were well integrated.					
6. I thought there was too much inconsistency in this system.					
7. I would imagine that most people would learn to use this system very quickly.					
8. I found the system very cumbersome to use.					
9. I felt very confident using the system.					
10. I needed to learn a lot of things before I could get going with this system.					
