# Supplementary material for: StatXFinder: a web-based self-directed tool that provides appropriate statistical test selection for biomedical researchers in their scientific studies
Source: Springerplus. 2015 Oct 22;4:633. doi: 10.1186/s40064-015-1421-9 (PMC4627976; doi:10.1186/s40064-015-1421-9)
Supplement: Supplementary file 3 — 10.1186/s40064-015-1421-9 User satisfaction questionnaire. [file 40064_2015_1421_MOESM3_ESM.pdf]

Questionnaire No:

A-USER INFORMATION

1. Academic Title:

2. Gender: □ Female □ Male

3. Age:

4. Level of computer use skills. □ Expert □ Advanced □ Average □ Elementary □ Beginner

5. Level of English proficiency. □ Proficient □ Advanced □ Intermediate □ Elementary □ Beginner

6. Level of statistics knowledge. □ Expert □ Advanced □ Average □ Elementary □ Beginner

7. Have you ever conducted a statistical analysis using a statistical software package? □ Yes □ No

B-SATISFACTION QUESTIONAIRE:

1. What features of the software do you like? Please specify with reasons?

2. What features of the software do you not like? Please specify with reasons?

3. Which parts of the software, if any, should be improved?

4. Is there any feature to be added to the software? Please specify.

5. Is there any feature to be removed from the software? Please specify.

6. Would you recommend this software to others?

□ Yes □ No

7. Please specify your overall recognition, evaluating the software as a whole.

□ I did not like at all. □ I did not like. □ No idea □ I like it. □ I like it very much.

Thank you for participating in the questionnaire.
