# Supplementary material for: StatXFinder: a web-based self-directed tool that provides appropriate statistical test selection for biomedical researchers in their scientific studies
Source: Springerplus. 2015 Oct 22;4:633. doi: 10.1186/s40064-015-1421-9 (PMC4627976; doi:10.1186/s40064-015-1421-9)
Supplement: Supplementary file 4 — 10.1186/s40064-015-1421-9 The list of recommended statistical tests by StatXFinder. [file 40064_2015_1421_MOESM4_ESM.pdf]

| No: | Statistical Test Recommendations |
|---|---|
| 1 | Use one sample z test |
| 2 | Use one sample t test |
| 3 | Use one sample chi-square test for variations while your inference concerning population Standard Deviation (Caution: This test is very sensitive to non-normality) |
| 4 | Use one sample binomial test with exact methods |
| 5 | Use one sample binomial test with normal theory methods |
| 6 | Use one sample Poisson test |
| 7 | Use Chi square Goodness of Fit Test to test the goodness of fit of another probability model or use nonparametric methods such as One-Sample Median Test to compare the sample median and a hypothesized value or use Runs test to determine the randomization of the data set. |
| 8 | Use two sample t test with equal variances (unpaired (independent) t-test) |
| 9 | Use two sample t test with unequal variances (Welch's t test) |
| 10 | Use paired t test |
| 11 | Use the Mantel-Haenszel test. |
| 12 | Use two sample test for binomial proportions or 2x2 contingency table methods. |
| 13 | Use the Mantel Extension test. |
| 14 | Use chi-square test for trend. |
| 15 | Use chi-square test for heterogeneity for 2xk tables |
| 16 | Use chi-square test for heterogeneity for RxC tables (for R>2 and C>2) |
| 17 | Use Fisher's exact test |
| 18 | Use McNemar's test |
| 19 | Use log-rank test |
| 20 | Use Cox proportional hazards model if you Interested in effects of several risk factors on survival. |
| 21 | Use Mann-Whitney U test (Wilcoxon rank sum test) |
| 22 | Use RxC contingency table methods |
| 23 | Use another underlying distribution or use nonparametric methods such as Kruskal-Wallis test or Mood's Median Test |
| 24 | Use simple linear regression |
| 25 | Use Pearson correlation methods |
| 26 | Use Rank Correlation Methods such as Spearman's rho |
| 27 | Use Nonparametric ANOVA (Kruskal-Wallis test) |
| 28 | Use analysis of covariance |
| 29 | Use One-way ANOVA.If all the means are found equal, no need to use multiple comparisons (post-hoc) tests. If all the means are found not equal, use multiple comparisons (post-hoc) tests. If you comparing groups with a control group, use Dunnett test. If not, use LSD. (least significant difference), Bonferroni, Sidak, Scheffe. , R-E-G-W F (Ryan-Einot-Gabriel-Welsch F test), R-E-G-W Q. (Ryan-Einot-Gabriel-Welsch range test), S-N-K. (Student-Newman-Keuls), Tukey, Tukey's b, Duncan, Hochberg's GT2, Gabriel, Waller-Duncan. |
| 30 | Use Welch's ANOVA. If all the means are found equal, no need to use multiple comparisons (post-hoc) tests. If all the means are not found equal, use multiple comparisons (post-hoc) tests such as Tamhane's T2, Dunnett's T3, Games-Howell,Â Dunnett's C. |
| 31 | Use Welch's ANOVA or Brown Forsythe test. If all the means are found equal, no need to use multiple comparisons (post-hoc) tests. If all the means are not found equal, use multiple comparisons (post-hoc) tests such as Tamhane's T2, Dunnett's T3, Games-Howell,Â Dunnett's C. |
| 32 | Use two way ANOVA. If all the means are found equal, no need to use multiple comparisons (post-hoc) tests. If all the means are not found equal, use multiple comparisons (post-hoc) tests. If you comparing groups with a control group, use Dunnett test. If not, use LSD. (least significant difference), Bonferroni, Sidak, Scheffe. , R-E-G-W F (Ryan-Einot-Gabriel-Welsch F test), R-E-G-W Q. (Ryan-Einot-Gabriel-Welsch range test), S-N-K. (Student-Newman-Keuls), Tukey, Tukey's b, Duncan, Hochberg's GT2, Gabriel, Waller-Duncan. |
| 33 | Use contingency table methods such as Odds Ratios and Relative Risk Ratios |
| 34 | Use Kappa statistic |
| 35 | Use Bland-Altman plot, if you are interested in comparison of method. If you are interested in comparison of observers, use Intraclass Correlation Coefficient |

| | |
|---|---|
| 36 | Use ROC Curve |
| 37 | Use linear multiple regression method with "Enter", “Stepwise”, "Forward" or "Backward” variable selection option. |
| 38 | Use test of trend for incidence rates over more than two exposure groups (with weighted regression approach). |
| 39 | Use log-rank test |
| 40 | Use Cox proportional hazards model if you Interested in effects of several risk factors on survival. |
| 41 | Use multiple logistic regression method (Binary Logistic Regression) |
| 42 | Use Repeated Measures ANOVA (analysis of variance) |
| 43 | Use Friedman test if data is ranked or scored, or measured. Use Cochrane Q if two possible outcomes (binomial data) are exists. |
| 44 | Use Wilcoxon signed rank test, Sign test |
| 45 | Use Shapiro-Wilk test or One Sample Kolmogorov-Smirnov test or Anderson-Darling test or Cramer-von Mises test to check the normality assumption of the data. |
| 46 | Use Bartlett Test or Levene Test to compare homogenity of variances. |
| 47 | Use two-sample F test to compare variances (Caution: This test is very sensitive to nonnormality). |
| 48 | Use Ordered Logistic Regression |
| 49 | Use higher-way ANOVA. If all the means are found equal, no need to use multiple comparisons (post-hoc) tests. If all the means are not found equal, use multiple comparisons (post-hoc) tests. If you are comparing groups with a control group, use Dunnett test. If not, use LSD. (least significant difference), Bonferroni, Sidak, Scheffe. , R-E-G-W F (Ryan-Einot-Gabriel-Welsch F test), R-E-G-W Q. (Ryan-Einot-Gabriel-Welsch range test), S-N-K. (Student-Newman-Keuls), Tukey, Tukey's b, Duncan, Hochberg's GT2, Gabriel, Waller-Duncan. |
| 50 | Use rank correlation methods such as Kendall's tau or Spearman's rho for ordinal data, contingency coefficient on chi-square table (chi-square test of independence) for nominal or ordinal data. |
| 51 | Warning! You should know the distribution of your data to make decision to choose appropriate statistical test according to your data. Please go to the starting point of the algorithm. |
| 52 | Use ANOVA (Analysis of Variance) after checking the assumptions of the method. If all the means are found equal, no need to use multiple comparisons (post-hoc) tests. If all the means are found not equal, use multiple comparisons (post-hoc) tests. If you are comparing groups with a control group, use Dunnett test. If not, use LSD. (least significant difference), Bonferroni, Sidak, Scheffe. , R-E-G-W F (Ryan-Einot-Gabriel-Welsch F test), R-E-G-W Q. (Ryan-Einot-Gabriel-Welsch range test), S-N-K. (Student-Newman-Keuls), Tukey, Tukey's b, Duncan, Hochberg's GT2, Gabriel, Waller-Duncan. |
| 53 | Use simple logistic regression |
| 54 | Use non-linear regression method or transform your data. |
| 55 | Use multiple non-linear regression method or transform your data. |
| 56 | Use one-sample test for incidence rates (A large-sample test based on the normal approximation to the Poisson distribution, when the expected number of events is equal or upper 10, and a small-sample test based on the exact Poisson probabilities). |
| 57 | Use two-samples test for stratified person-time data. |
| 58 | Use two-samples test for comparison of incidence rates. |
| 59 | Warning! Before predict one variable from another, the assumptions such as linear relationship between dependent and independent variables, statistical independence of the errors, homoscedasticity (constant variance) of the errors and normality of the error (residuals) distribution should be checked. |
