# Supplementary material for: StatXFinder: a web-based self-directed tool that provides appropriate statistical test selection for biomedical researchers in their scientific studies
Source: Springerplus. 2015 Oct 22;4:633. doi: 10.1186/s40064-015-1421-9 (PMC4627976; doi:10.1186/s40064-015-1421-9)
Supplement: Supplementary file 5 — 10.1186/s40064-015-1421-9 Decision questions and corresponding explanations. [file 40064_2015_1421_MOESM5_ESM.pdf]

No	Decision Questions	Explanations
1	Does your data set have only one variable?	Do you have only one variable to analyse, such as age or sex? (For instance, do you want to compare the mean value of a continuous "random variable" such as age with a known population mean?).
2	Is it a "one" sample (=group) problem?	Do you have only one sample? Do you want to analyze the features of a single sample? (In that case, you are not interested in two or more groups.)
3	Are you going to test the relationships between two variables?	Do you want to anaylse the relationship of two variables in your data set? In other words, do you want to know whether one variable changes with the other; whether variables are related; whether one causes the other?
4	Do you know the type of the distribution of data?	Does the distribution of the "random variable" in the data set correspond to a continuous probability distribution such as normal distribution, log normal distribution, Weibull distribution, exponential distribution or to a discrete probability distribution such as binomial distribution, geometric distribution, negative binomial distribution, hypergeometric distribution, Poisson distribution, uniform distribution.
5	Is it a "two" samples (=groups) problem?	Do you have two samples? Do you want to analyse the features of two distinct samples? (In that case, you are not interested in a single group or more than two groups.)
6	Are both of your variables continuous?	Do both variables under analysis have continuous natures?
7	Is your outcome (=dependent) variable continuous?	In cases where more than two variables are under analysis, is the "dependent variable" continuous in nature?
8	Does your data appear to be normally distributed (bell-shaped curve)?	Does the distribution of the continuous variable under analysis have the features of a normal distribution? Graphical and statistical analysis methods are used to examine whether the distribution of the data is normal or not. Note: Use Shapiro-Wilk test or One Sample Kolmogorov-Smirnov test or Anderson-Darling test or Cramér-von Mises test to check the normality assumption of the data.
9	Do you want to compare the means of more than two samples (=groups)?	Do you want to compare the means of more than two samples? For instance, comparing the mean monthly temperatures of three cities such as London, Paris and New York.
10	Do you want to predict one variable from another?	Do you want to predict one variable using another variable? For instance, using oestriol levels to predict the birth weight in pregnant women (Rosner, B. (2000). Fundamentals of Biostatistics, 5th Edition, Duxbury Press: USA, p. 425).
11	Is one variable continuous and the other one categorical?	Is one of the variables continuous in nature, while the other one is nominal, also referred as categorical?
12	Is the outcome (=dependent) variable ordinal?	In cases where more than two variables are under analysis, is the "dependent variable", which is thought to have an effect on other variables, ordinal?
13	Do you want to check the normality assumption of the data?	Do you want to question whether the distribution of the continuous variable under analysis has the features of normal distribution?
14	Do you want to make an inference concerning mean?	In cases when you cannot obtain information about the population under analysis, inferences about the population are made via the sample. Do you want to make inferences about population mean?
15	Does your data have a binomial distribution?	Does the distribution of the discrete variable under analysis have the features of the binomial distribution?
16	Do you want to make inferences concerning means?	In cases when you cannot obtain information about the population under analysis, inferences about the population are made via the sample. Do you want to make inferences including population means?
17	Are your samples (=groups) independent?	Are the samples under analysis independent? In other words the data groups you think that represent the population, independent? For instance, when pairwise observations such as pre-treatment vs. post-treatment are not taken, two samples are independent.
18	Do you want to compare observers / methods?	Do you want to analyze cases which require to compare the degree of association or reproducibility of response between assessments by experts/referees, between methods or between two measurements repeated on the same subject?
19	Do you have the time of events in your data?	Any set of the results under analysis is called an "event". Is the occurrence time or duration of these events significant, or is the existence of the event sufficient for the analysis? For instance, if the incidence of disease varies over time, the time of events is significant in the comparison of the number of disease events between two groups; e.g. time of events is important in testing the hypothesis that older smokers are less successful in giving up smoking (Rosner, B. (2000). Fundamentals of Biostatistics, 5th Edition, Duxbury Press: USA, p. 711).
20	Is at least one variable ordinal?	Is at least one variable under analysis measured via an ordinal scale? For instance, level of education, level of income, etc.
21	Do you know the standard deviation of the population?	Do you know the standard deviation value for the population from which the sample was taken?
22	Is the normal approximation valid?	Since it would be difficult to study with binomial distribution when sample size is large (in case sample size is greater than 30), normal approximation facilitates the analysis rather than an exact binomial distribution (Rosner, B. (2000). Fundamentals of Biostatistics, 5th Edition, Duxbury Press: USA, p. 138).

23	Does your data have a Poisson distribution?	Does the distribution of the discrete variable under analysis have the features of the Poisson distribution?
24	Do you have person-time data?	Was your data obtained from measurements combining person data and time data? For instance, you have data from a population from which some individuals (person) who developed lung cancer over a year (time).
25	Are both of your variables normally distributed (bell-shaped curve)?	Do the distributions of both variables under analysis have the features of normal distribution? Graphical and statistical analysis methods are used to examine whether the distribution of the data is normal or not. Note: Use Shapiro-Wilk test or One Sample Kolmogorov-Smirnov test or Anderson-Darling test or Cramér-von Mises test to check the normality assumption of the data.
26	Is there only one categorical variable which affects the continuous variable?	Do you want to analyse cases in which groups are compared with reference to a single categorical variable? For instance analysing lung functions with regard to smoking condition (non-smoker, passive smoker, low level smoker, mid-level smoker, heavy smoker, etc.) (Rosner, B. (2000). Fundamentals of Biostatistics, 5th Edition, Duxbury Press: USA, p. 551). If you would like to analyse another variable such as diet, in addition to smoking, then a two-way analysis would be used rather than a one-way analysis.
27	Do you want to test the association between two categorical variables?	Do you want to test the association between two categorical variables? For instance, analysing the myocardial infarction incidence in three years in women using and not using oral contraceptives.
28	Do incidence rates remain constant over time?	Does the incidence rate, which is defined as the ratio of individuals developing a disease between a certain period of time between two groups, one exposed to the disease and the other non-exposed, change in time? For instance, change of incidence of cancer as the age increases (Rosner, B. (2000). Fundamentals of Biostatistics, 5th Edition, Duxbury Press: USA, p. 678).
29	Is there a significant difference between the variances of two samples (=groups)?	Does the variances of two samples show a statistically significant difference when tested with F-test?
30	Are all expected values of observations in the contingency table are equal to or greater than 5?	Are the expected values of the observations in the contingency table, used in comparisons of discrete variable in studies where two independent samples are compared, equal to or greater than 5?
31	Do you have categorical data?	Do the variables under analysis comprise of nominal or ordinal data which do not have continuous measurement values such as age, height, etc.?
32	Does your outcome (=dependent) variable appear to be normally distributed (bell-shaped curve)?	Does the distribution of the continuous variable under analysis have the features of a normal distribution? Graphical and statistical analysis methods are used to examine whether the distribution of the data is normal or not. Note: Use Shapiro-Wilk test or One Sample Kolmogorov-Smirnov test or Anderson-Darling test or Cramér-von Mises test to check the normality assumption of the data.
33	Are there two categorical variables which affect the continuous variable?	Do you want to analyse cases in which groups are compared with reference to two distinct variables? For instance analysing lung functions with regard to smoking condition (non-smoker, passive smoker, low level smoker, mid-level smoker, heavy smoker, etc.) and diet (vegetarian, lacto-vegetarian and normal) (Rosner, B. (2000). Fundamentals of Biostatistics, 5th Edition, Duxbury Press: USA, p. 542).
34	Do you want to compare survival curves of two groups with limited control of covariates?	Do you have a variable that is associated with a limited number of "dependent variables" for comparing two survival curves?
35	Is it possible to create a 2x2 contingency table using the available data?	Is it possible to create a contingency table of two categorical variables, each of which has two levels?
36	Are there any other covariates related to the variable(s) which should be controlled?	The effect of other variables that could influence the relation between the variables under analysis should be controlled. For instance, the effect of smoking variable should be controlled, when analysing the relation between lung cancer and drinking (heavy drinker, 2 or more drinks a day); because smoking is a variable both related to lung cancer and drinking (Rosner, B. (2000). Fundamentals of Biostatistics, 5th Edition, Duxbury Press: USA, p. 591).
37	Is assumption of homogeneity of variance met?	Are the population variances equal to each other? In other words, are the variances homogenous? Do the populations have homoscedasticity/homogeneity of variance? Note: Test using Bartlett Test, Levene Test or Brown-Forsythe Test for checking the homogenity of the population variance.
38	Is it possible to create a 2xk contingency table using the available data?	Is it possible to create a contingency table of two categorical variables with two and k levels?
39	Do you want to conduct an analysis of trend, such as an increase or a decrease, over k binomial proportions?	Are you conducting a study which analyses whether there is a trend in binomial rates, such as an increase or decrease in success? For instance, analysing breast cancer development in the first birth age group with regard to case-control groups (Rosner, B. (2000). Fundamentals of Biostatistics, 5th Edition, Duxbury Press: USA, p. 393).
40	Did you check the assumptions of data?	Before predicting one variable from another, the assumptions such as linear relationship between dependent and independent variables, statistical independence of the errors, homoscedasticity (constant variance) of the errors and normality of the error (residuals) distribution should be checked.
41	Is the linearity assumption met?	To use linear models, the linear relationship between dependent and independent variables should be met.

42	Is confounding present?	Is there a variable which is associated with both disease and the exposure variable? The effect of other variables that could influence the relation between the variables under analysis should be controlled. For instance, the effect of smoking variable should be controlled, when analysing the relation between lung cancer and drinking (heavy drinker, 2 or more drinks a day); because smoking is a variable both related to lung cancer and drinking (Rosner, B. (2000). Fundamentals of Biostatistics, 5th Edition, Duxbury Press: USA, p. 591).
43	Do you want to predict one binary variable from another continuous variable?	Do you want to predict one variable which has two possible values (0 or 1) using another variable? For instance, suppose that you want to determine whether there is a relationship between sand grain size and the presence or absence of spider. Spider presence or absence is the dependent variable; because sand grain size affecting presence of spiders.
44	Do you want to quantify the diagnostic accuracy of the test?	If you want to compare the test results with a final true diagnosis, it means accuracy of the test.
